# Daratumumab augments alloreactive natural killer cell cytotoxicity towards CD38+ multiple myeloma cell lines in a biochemical context mimicking tumour microenvironment conditions

**DOI:** 10.1007/s00262-018-2140-1

**Published:** 2018-03-02

**Authors:** Niken M. Mahaweni, Gerard M. J. Bos, Constantine S. Mitsiades, Marcel G. J. Tilanus, Lotte Wieten

**Affiliations:** 1 Division of Hematology, Department of Internal Medicine, Maastricht University Medical Center+, Maastricht, The Netherlands; 2 Department of Transplantation Immunology, Tissue Typing Laboratory, Maastricht University Medical Center+, P.O. Box 5800, 6202 AZ Maastricht, The Netherlands; 3 Department of Medical Oncology, Dana-Farber Cancer Institute, Boston, MA USA; 4 Department of Medicine, Harvard Medical School, Boston, MA USA

**Keywords:** Alloreactive NK cells, Multiple myeloma, Daratumumab, Tumor microenvironment, ADCC

## Abstract

Natural killer (NK) cell-based immunotherapy is a promising novel approach to treat cancer. However, NK cell function has been shown to be potentially diminished by factors common in the tumor microenvironment (TME). In this study, we assessed the synergistic potential of antibody-dependent cell-mediated cytotoxicity (ADCC) and killer immunoglobin-like receptor (KIR)-ligand mismatched NK cells to potentiate NK cell antitumor reactivity in multiple myeloma (MM). Hypoxia, lactate, prostaglandin E2 (PGE2) or combinations were selected to mimic the TME. To investigate this, NK cells from healthy donors were isolated and NK cell ADCC capacity in response to MM cells was assessed in flow cytometry-based cytotoxicity and degranulation (CD107a) assays in the presence of TME factors. Hypoxia, lactate and PGE2 reduced cytotoxicity of NK cells against myeloma target cells. The addition of daratumumab (anti-CD38 antibody) augmented NK-cell cytotoxicity against target cells expressing high CD38, but not against CD38 low or negative target cells also in the presence of TME. Co-staining for inhibitory KIRs and NKG2A demonstrated that daratumumab enhanced degranulation of all NK cell subsets. Nevertheless, KIR-ligand mismatched NK cells were slightly better effector cells than KIR-ligand matched NK cells. In summary, our study shows that combination therapy using strategies to maximize activating NK cell signaling by triggering ADCC in combination with an approach to minimize inhibitory signaling through a selection of KIR-ligand mismatched donors, can help to overcome the NK-suppressive TME. This can serve as a platform to improve the clinical efficacy of NK cells.

**Electronic supplementary material:**

The online version of this article (10.1007/s00262-018-2140-1) contains supplementary material, which is available to authorized users.

## Introduction

NK cell-based immunotherapy is a promising therapeutic approach to treat cancer. NK cells selectively target cancer cells and induce potent anti-cancer responses while sparing non-cancer cells [1]. A potential obstacle to NK cell therapy is the suppressive tumor microenvironment (TME). The TME contributes to the acquisition of therapy-resistant cancer cells posing a potential limitation for any anti-cancer therapy including immunotherapy [2, 3]. TME factors (TMEFs), for example: hypoxia [4, 5]; prostaglandin E2 (PGE2) [6]; lactate [7, 8]; galectin-3 [9]; platelet-derived growth factor [10]; transforming growth factor β1 [11]; as well as the presence of other immune cells such as myeloid-derived suppressor cells [12, 13], have also been shown to contribute to diminished NK cell antitumor reactivity. Hence, to further optimize the clinical response of adoptive NK cell therapy, clinically applicable strategies to potentiate the NK cell antitumor response, which facilitate the NK cell function in the suppressive TME, are warranted.

The activation of NK cells is determined by the signaling balance between inhibitory and activating NK cell receptors. Either maximizing activating signaling or reducing inhibitory signaling would be a feasible strategy to improve NK cell efficacy. Activating receptors, typically bind to stress-induced ligands expressed by diseased or transformed cells. The most potent activating NK cell receptor is CD16, a low-affinity Fc receptor which binds to the Fc portion of an IgG antibody triggering antibody-dependent cell-mediated cytotoxicity (ADCC) [14]. The current availability of a large array of clinical-grade monoclonal antibodies (mAbs) to treat cancer provides a potent opportunity to enhance the NK cell anti-cancer response via the ligation of CD16 to a cancer antigen-specific antibody subsequently resulting in cancer cell death [15]. The ADCC effect of different therapeutic mAbs such as rituximab, obinutuzumab, trastuzumab, and cetuximab has been described to be mainly NK cell-dependent [16]. Nijhof et al. [17] also reported that daratumumab, a more recently engineered mAb against CD38, could trigger NK cell ADCC activity against multiple myeloma (MM) cells. Moreover, one study reported that rituximab could trigger ADCC even under 1% O_2_ albeit at a lower level than under 20% O_2_ [5]. Inhibitory receptors such as killer immunoglobulin-like receptors (KIRs) interact with human leukocyte antigen (HLA) class I molecules, expressed on the membrane of nearly all healthy cells, to prevent autoreactivity. Approaches to minimize signaling via strongly inhibitory NK receptors, such as KIRs and NKG2A, and to reduce the activation threshold for NK-cell activation might be especially crucial in situations where there are already many inhibitory signals present. We recently demonstrated that, also under hypoxic conditions, KIR-ligand mismatched NK cells were more potent effector cells against MM than KIR-ligand matched NK cells [18].

Inhibitory KIRs and CD16 are both primarily expressed on CD56dim cells and previous studies showed that a KIR-ligand interaction might negatively influence NK cell-mediated ADCC [16, 19, 20]. In this study, we, therefore, hypothesized that the combination of triggering ADCC and KIR-ligand mismatching could provide a potent platform to potentiate the NK cell antitumor response in the TME. To study this hypothesis, we used daratumumab to evaluate the NK cell-mediated ADCC response to MM cells in the presence of a selected combination of TME factors. These selected TME factors hypoxia, lactate and PGE2 are frequently found in the TME of many tumors and have been described to hamper NK cell antitumor response. In addition, we determined whether KIR-ligand mismatched NK cells were more potent than matched NK cells under these conditions. For the experiments, we used IL-2 activated NK cells to resemble the clinical situation where ex vivo (IL-2) activated NK cells will be infused into cancer patients.

## Materials and methods

### Cell lines and culture

The K562 cell line was cultured in IMDM and 10% fetal calf serum (FCS). The OPM-2, UM-9 RPMI8226/s cell lines were cultured in RPMI1640 and 10% FCS, the L363 cell line was cultured in RPMI1640 and 15% FCS, the JJN-3 cell line was cultured in 40% IMDM and 40% low glucose DMEM with 20% FCS. All cell culture media were supplemented with 100 U/mL penicillin (Gibco) and 100 µg/mL streptomycin (Gibco). K562 and RPMI8226/s were obtained from American Type Culture Collection (ATCC, Rockville, MD, USA). OPM-2, L363, and JJN-3 were obtained from Deutsche Sammlung von Mikroorganismen und Zellkulturen (DSMZ GmbH, Braunschweig, Germany). UM-9 was a gift from Dr. A. Martens, Vrije Universiteit Medisch Centrum (VUMC), The Netherlands. All culture media were from Gibco, Breda, The Netherlands and FCS was produced by Greiner Bio-One International, GmbH. All cell lines were cultured at 37 °C in humidified air containing 5% CO_2_ with 21% O_2_ (Sanyo MCO-20AIC, Sanyo Electric Co, Japan).

### NK cell isolation and activation

NK cells were isolated from fresh blood derived from healthy donors after signing informed consent or from healthy donor’s HLA-typed buffy coats. Donors with an HLA-C1+C2+Bw4+ genotype were selected. The use of buffy coats, being a by-product of a required Medical Ethical Review Committee (METC) procedure, does not need ethical approval in The Netherlands under the Dutch Code for Proper Secondary Use of Human Tissue. These buffy coats were anonymous, and the individuals from whom the samples originated did not object to their use. PBMCs were obtained by density gradient centrifugation of the donor sample using lymphoprep (Axis-Shield). NK cells were subsequently isolated by negative selection with an NK cell isolation kit using MACS beads and columns according to manufacturer’s protocol (Miltenyi Biotec, GmbH). For short-term activation, NK cells were cultured in RPMI-1640 medium (Gibco) supplemented with 10% fetal calf serum (Greiner Bio-One), 100 U/mL penicillin (Gibco) and 100 µg/mL streptomycin (Gibco) at 37 °C in humidified air containing 5% CO_2_ with 21% O_2_ (Sanyo MCO-20AIC, Sanyo Electric Co, Japan). NK cells were activated overnight with 1000 IU/mL recombinant human IL-2 (Proleukin, Novartis).

### CD107a degranulation assay

To assess NK cell degranulation against MM target cells (tumor cells), CD107a expression on NK cells was analyzed using flow cytometry. Target cells were plated in 24 wells plate at a concentration of 2 × 10^6^ cells/mL per well and incubated overnight at 37 °C in humidified air containing 5% CO_2_ with 21% O_2_ (Sanyo MCO-20AIC, Sanyo Electric Co, Japan) or 0.6% O_2_ (Invivo_2,_ 1000 Ruskinn Technology Ltd, Bridgend, UK). Prior to the assay, IL-2 activated NK cells were harvested, washed, and subjected to 1-h incubation with either 50 mM sodium l-lactate (Sigma), 100 ng/mL prostaglandin (Sigma), or medium. Target cells were pre-incubated for 30 min with 1 µg/mL daratumumab (Genmab) or trastuzumab (Roche) or, as a control, with medium at 21% O_2_ (ambient air) or 0,6% O_2_ (hypoxia). TMEF-exposed NK cells were then, in duplicate wells, co-cultured with the target cells in 1:1 effector:target ratio and 2 µL anti-CD107a-Horizon V450 (H4A3, BD Biosciences) was added per well. After 1 h of co-culture, monensin (BD Biosciences) was added. After another 3 h, the plate was placed on ice to stop the reaction. Cells were then stained on ice with anti-CD3-APC/H7 (SK7, BD Biosciences), anti-CD56-PeCy7 (B159, BD Biosciences), anti-KIR2DL1-APC (143211, R&D), anti-KIR2DL2/3/S2-PE (DX27, Miltenyi Biotec), anti-KIR3DL1-FITC (DX9, Miltenyi Biotec), and anti-NKG2A-PC5.5 (Z199, Beckman Coulter).

### Analysis of KIR-ligand matched and mismatched NK cells

Using Luminex-SSO, we determined the genotypic expression of the HLA class I epitopes of UM9 (C1+C2−Bw4−) and RPMI8226/s (HLA C1+C2+Bw4−) at the genomic level. KIR-ligand matched NK cells for UM9 were KIR2DL2/3+ while for RPMI8226/s they were KIR2DL2/3+, KIR2DL1+, or the combination of KIR2DL2/3+ and KIR2DL1+. KIR-ligand mismatched NK cells for UM9 were KIR2DL1+, KIR3DL1+ or the combination of KIR2DL1+ and KIR3DL1+, while for RPMMI8226/s they were KIR3DL1+.

### Cytotoxicity assay

The NK cell cytotoxic potential against tumor cells was determined in a 4-h flow cytometry-based assay. Tumor cells were labeled using CellTracker™CM-DiI Dye (Molecular Probes™, USA) and were incubated overnight at 37 °C in humidified air containing 5% CO_2_ with 21% O_2_ (ambient air) (Sanyo MCO-20AIC, Sanyo Electric Co, Japan) or 0.6% O_2_ (hypoxia) (Invivo_2,_ 1000 Ruskinn Technology Ltd, Bridgend, UK). Prior to the assay, IL-2 activated NK cells were harvested and washed followed by 1-h incubation with either 50 mM sodium lactate (Sigma) or 100 ng/mL PGE2(Sigma) or medium. Tumor cells were pre-incubated for 30 min with 1 µg/mL daratumumab, trastuzumab, rituximab (Roche), or medium under ambient air or hypoxia. After pre-incubation of with TMEFs, NK cells were co-cultured with labeled tumor cells in 1:1 effector:target ratio for 4 h in duplicates. After 4 h, dead DiI-labeled tumor cells were measured with Live/Dead^®^ Fixable Aqua Dead Cell Stain Kit (Molecular Probes™, USA). Specific cytotoxicity was determined by the equation: (% dead tumor cells − % spontaneous tumor cell death)/(100% − % spontaneous tumor cell death) × 100.

### Compartment specific-bioluminescence (CS-BLI) cyototoxicity assay

The assay was performed as described previously [21]. In short, luciferase positive cell lines RPMI8226/s, JJN3, and L363 were plated in optical 96-well plates (Corning) at 4000 or 6500 cells per well. NK cells, pre-incubated for 30 min with either lactate or PGE2, were added at a 1:5 effector:target ratio. After 24 h of co-culture, luciferin (Xenogen Corp) was added, and plates were incubated for an additional 30 min at 37 °C followed by immediate measurement of the bioluminescence using a Luminoskan (Labsystems). The percentage tumor cell killing was calculated by: 100% − (CS-BLI signal with NK cells/CS-BLI signal without NK cells) × 100%.

### Flow cytometry

Expression of MM specific antigens such as CD38-PE (BD Biosciences) and the above mentioned NK cell receptors was measured using flow cytometry. Cells were washed with PBS (Gibco) and stained first for dead cells using Live/Dead^®^ Fixable Aqua Dead Cell Stain Kit (Molecular Probes™, USA) for 30 min on ice in the dark. Cells were further washed with FACS buffer (PBS, 1% FCS) and stained with antibodies for 30 min on ice in the dark. All flow cytometric analyses were performed with BD FACS Canto II. Data were analyzed with FlowJo 10.1r5 64 bit software.

### Statistical analysis

All statistical analysis was performed with GraphPad Prism V software (Graphpad Software Inc, San Diego, CA, USA) using two-tailed non-parametric *t* test with repeated measure (Wilcoxon signed rank test). * indicates a p value of < 0.05.

## Results

### The tumor microenvironmental factors lactate and PGE2 can inhibit NK cell cytotoxicity against MM cells

**Fig. 1:**
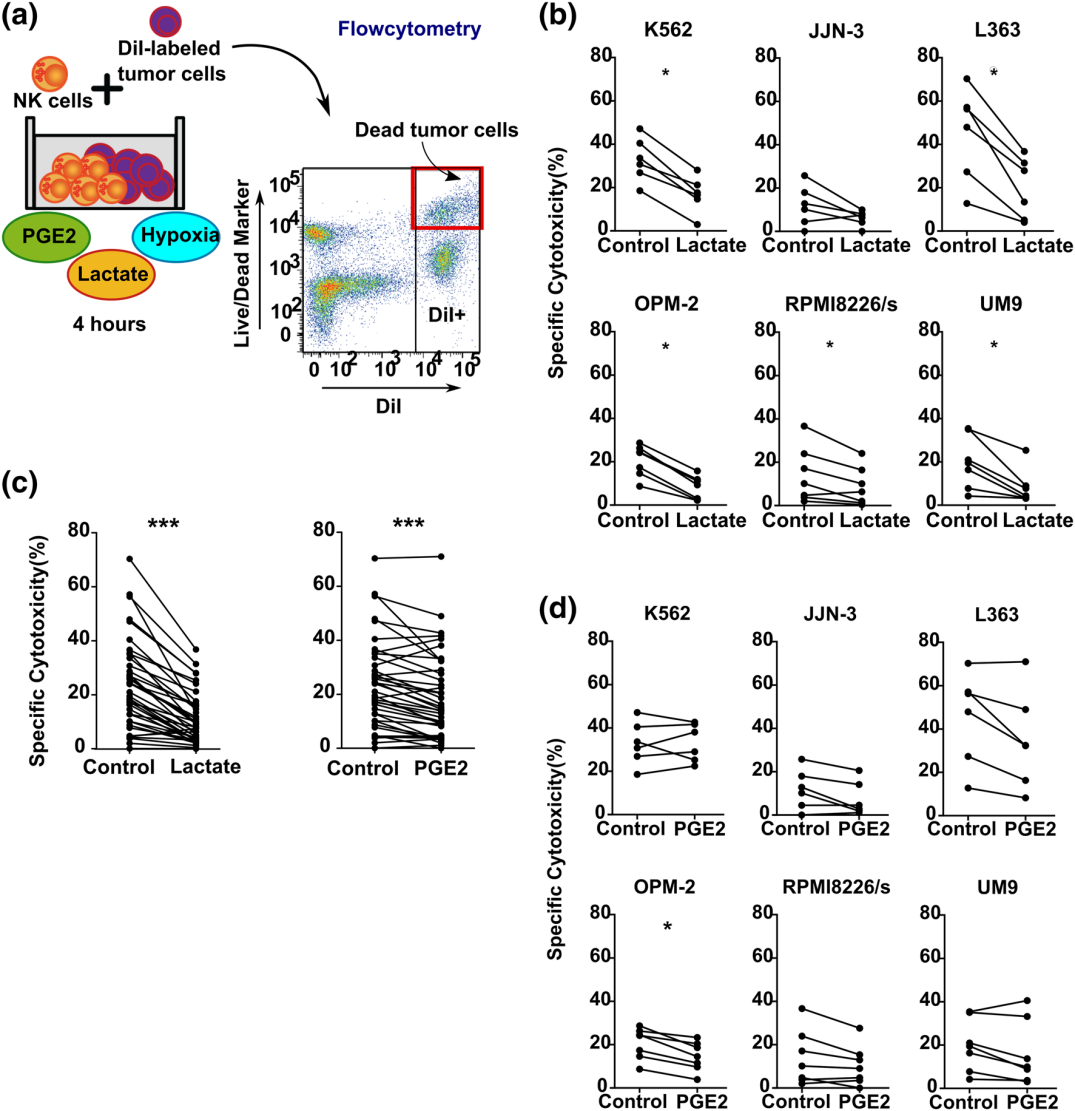
Analysis of the effect of combinations of tumor microenvironmental factors on the antitumor capacity of IL-2 activated NK cells. **a** Summary of the experimental set up: blood-derived NK cells were activated with IL-2 overnight. The following day, NK cells were washed and incubated for 1 h with either PGE2 or lactate followed by a 4-h cytotoxicity assay with DiI-labeled tumor cells that had been overnight incubated under hypoxia (0.6% O_2_). **b**–**d** Specific cytotoxicity of NK cells against K562, JJN-3, L363, OPM-2, RPMI8226, or UM9 cell lines under hypoxia without (control) or with lactate (**b**) or PGE2 (**c**). Data in b and c are from *n* = 6 different NK cell donors (every dot represents one donor). **d** Data from all cell lines used in **b** and **c** were pooled and statistical analysis was performed on pooled data. *p < 0.05, ***p < 0.0001

To study the effect of combinations of TMEFs on NK cell function, we used co-cultures of IL-2 activated primary NK cells with either MM cell lines or the HLA class I deficient K562 line. Previous studies observed that lactate and PGE2 concentrations of up to 40 mM (lactate) and 50 ng/mL (PGE2) could be found in tumors [22, 23]. To determine the NK cell potentiating effect of antibodies in a severely suppressive TME, we performed a dose titration (supplementary Fig. 1) and selected 50 mM lactate and 100 ng/mL PGE2 as concentrations to combine with hypoxia. As expected from our previous study [4], hypoxia (0.6% O_2_) alone did not influence cytotoxicity of IL-2 activated NK cells against all cell lines tested when compared to ambient air (21% O_2_) conditions (supplementary Fig. 2). However, the combination of hypoxia and lactate reduced NK cell cytotoxicity ranging between a 1.63 fold (for RPMI8226/s) to a 2.61-fold reduction (for OPM-2) (Fig. 1b). The average fold reduction of NK cell cytotoxicity for all cell lines together was 2.28-fold (*p* < 0.0001, Fig. 1d). The effect of the combination of hypoxia and PGE2 was less profound than the combination of hypoxia and lactate. It did not reduce NK cell cytotoxicity against K562. For the MM cell lines, the reduction ranged between 1.23-fold reduction (for UM-9) and 1.58-fold reduction (for JJN-3) (Fig. 1c). The average fold reduction of NK cell cytotoxicity against all cell lines tested was 1.26 (*p* < 0.0001, Fig. 1d). To exclude the possibility that the inhibition was due to an increase in NK cell death caused by the TMEFs itself, we tested the viability of NK cells which demonstrated no differences in the percentage of dead NK cells in the presence of TMEFs (supplementary Fig. 3).

### Triggering ADCC with daratumumab can augment NK cell antitumor reactivity in the presence of single or combinations of TMEFs

To investigate whether ADCC-triggering antibodies (daratumumab, trastuzumab, rituximab) could potentiate the NK cell antitumor response in the presence of TMEFs, we performed cytotoxicity assays with or without incubation of the tumor cells with antibodies. In the presence of hypoxia alone, all three antibodies could boost NK-cell cytotoxicity when NK cells were co-cultured with cell lines expressing the target antigens (supplementary Fig. 4). We selected daratumumab to further evaluate the ADCC effect in the presence of combinations of TMEFs.

**Fig. 2:**
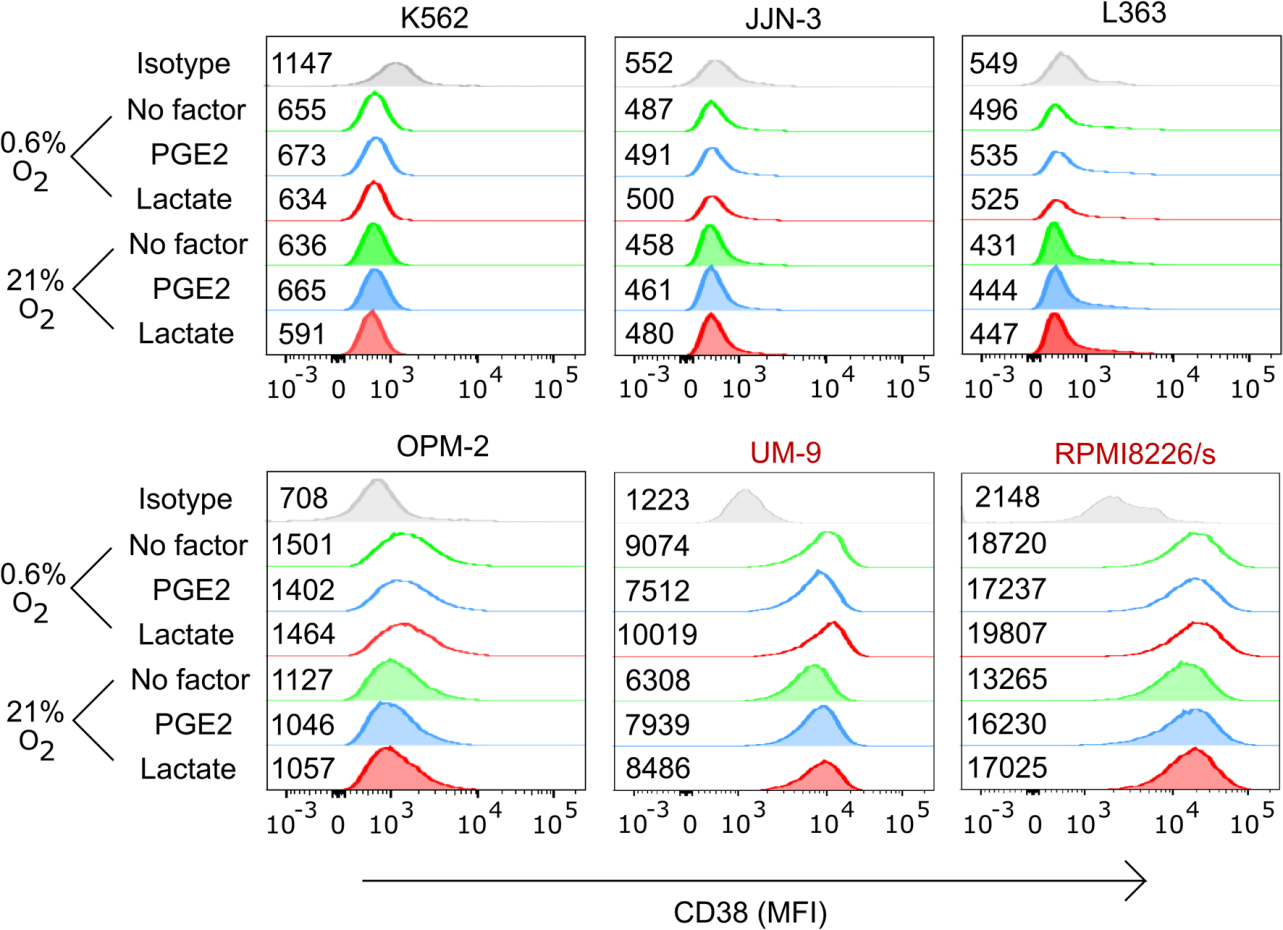
Analysis of the effect of hypoxia, lactate, PGE2 or combination thereof on CD38 expression levels of multiple myeloma cell lines. UM9, RPMI8226, OPM-2, JJN-3, L363 and K562 cell lines were cultured overnight under 0.6 or 21% O_2_ followed by a 4 h incubation with or without PGE2 or lactate. CD38 expression was determined using flow cytometry. The median fluorescence index (MFI) is indicated next to each histogram. Figure is representative of three independent experiments. Cell lines having high CD38 expression are denoted in red

**Fig. 3:**
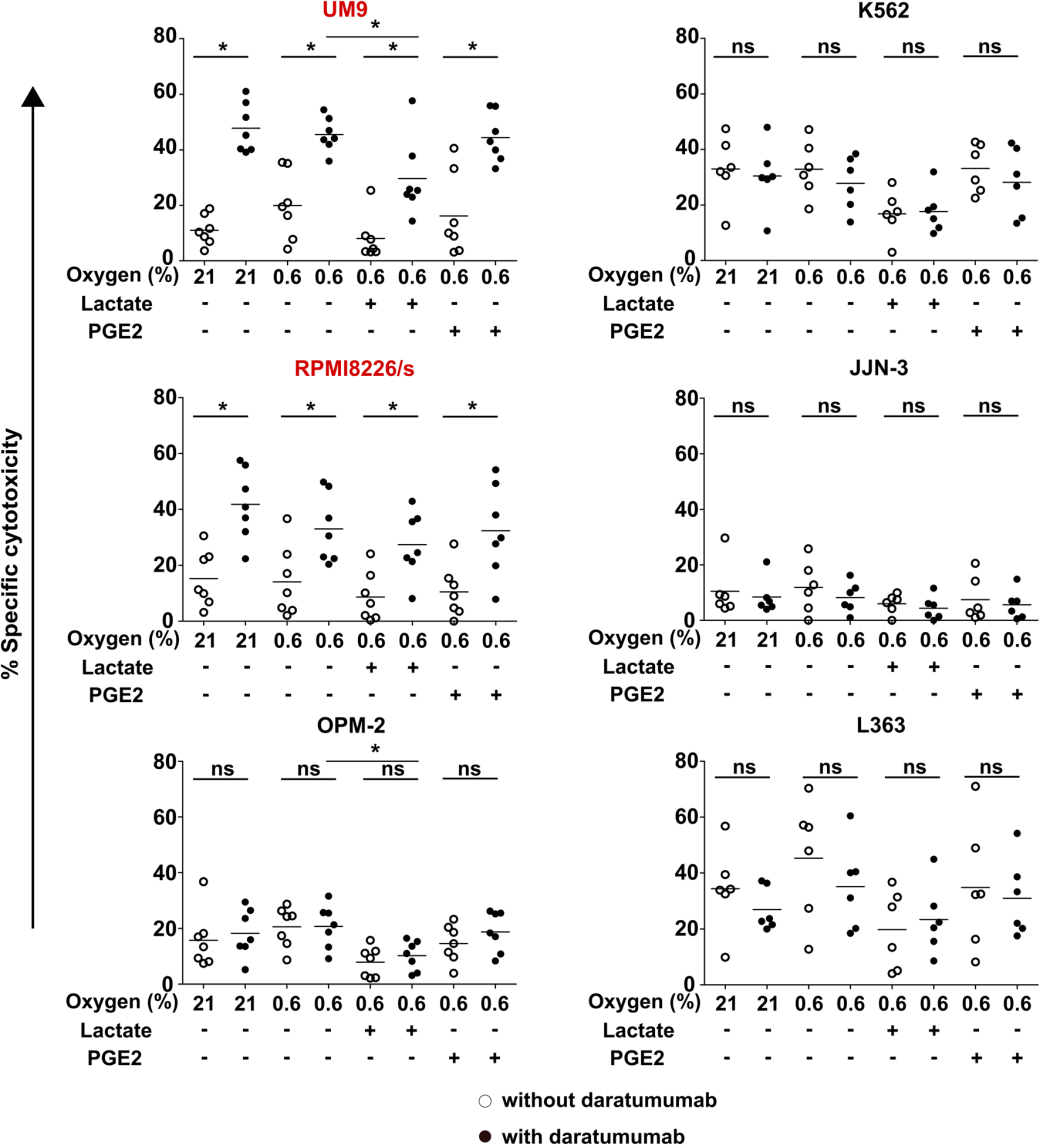
Effect of daratumumab on NK cell killing of CD38-high cells under suppressive TMEF. DiI-labeled UM9, RPMI8226, OPM-2, K562, JJN-3 and L363 cells were incubated overnight at 0.6 or 21% O_2_. The next day, tumor cells were pre-incubated with daratumumab while overnight IL-2 activated NK cells were pre-incubated with PGE2 or lactate followed by co-culture in a cytotoxicity assay. Graphs show specific cytotoxicity data. White dots = without daratumumab, black dots = with daratumumab. *p < 0.05, *n* = 6 independent donors (K562, JJN-3, and L363) or 7 donors (UM9, RPMI8226, OPM2). Cell lines having high CD38 expression are denoted in red

For daratumumab to trigger ADCC, the CD38 antigen expression on the cell surface must persist under TME conditions. We therefore determined CD38 expression on myeloma cells upon culture with TMEFs. Flow cytometry showed that RPMI8226/s and UM9 were high in CD38 expression, while OPM-2 was low in CD38. L363, JJN-3, and K562 were CD38-negative. Moreover, CD38 expression levels remained constant in the presence of TMEF (Fig. 2). In subsequent cytotoxicity assays, we showed that daratumumab enhanced NK cell cytotoxicity against the CD38-high MM cell lines, from 20 to 45% for UM9 and from 14 to 33% for RPMI8226/s (Fig. 3). Importantly, daratumumab enhanced the NK cell anti-MM response in the presence of all tested combinations of TMEF. For UM9, under hypoxic and lactate conditions, the increase in ADCC was lower compared with the increase in the hypoxia only condition (*p* = 0.0023). Daratumumab did not trigger NK cell-mediated ADCC under any of the conditions where NK cells were co-cultured with CD38low OPM-2 cells, suggesting that the expression level of CD38 on the target cells was important for the potential of the antibody to induce NK cell-mediated ADCC. As expected, daratumumab also did not enhance or reduce the killing of the CD38 negative cell lines JJN-3, L363, K562.

## Daratumumab induces NK cell death

**Fig. 4:**
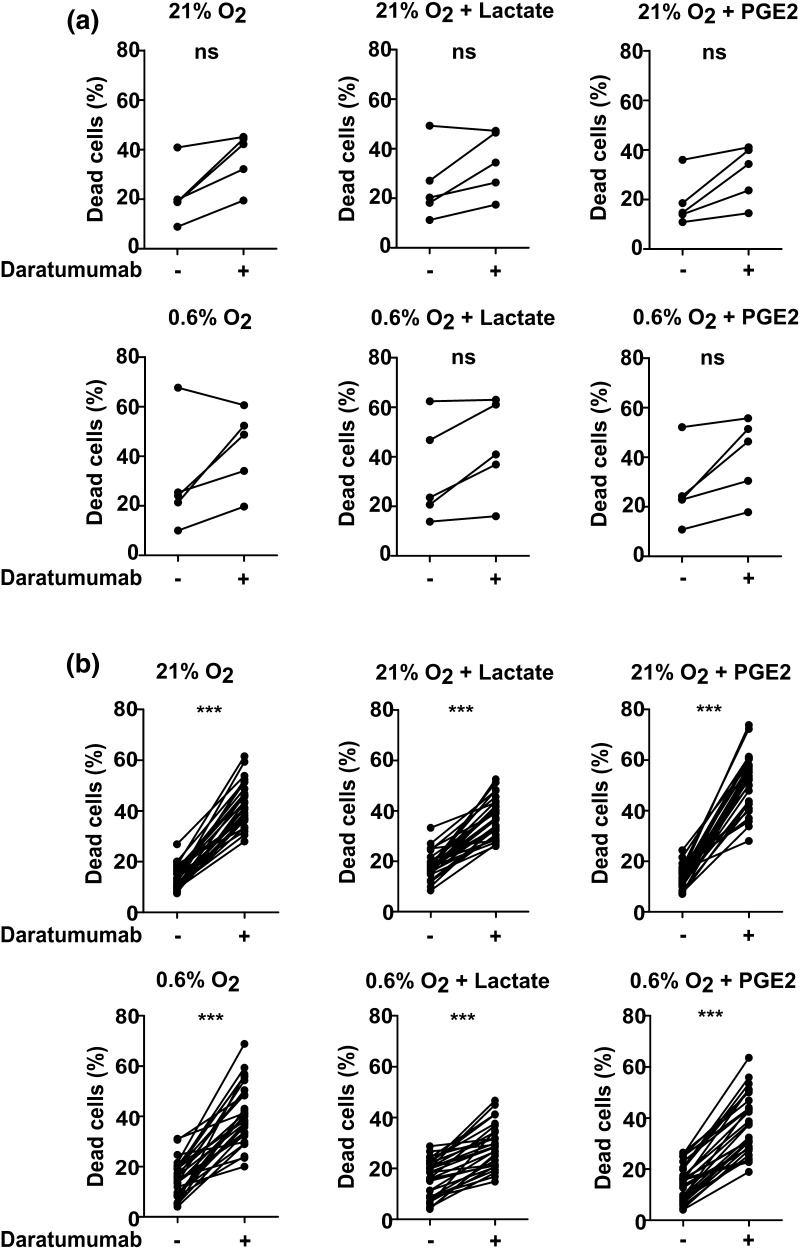
NK cell death in the absence or presence of daratumumab. **a** IL-2 activated NK cells were incubated with daratumumab. After 4 h, NK cell death was determined by flow cytometric analysis of a life-death marker. *n* = 5 independent donors, every dot indicates one donor. **b** Incubation of IL-2 activated NK cells with daratumumab and tumor cell lines (K562, JJN-3, L363, OPM-2, UM9 or RPMI8226/s), *n* = 5 independent donors, every dot indicates one donor. For graphs in **b**, NK cells from each of the five donors were incubated with all six cell lines. Data from all cell lines were collectively plotted in one graph. ***p < 0.0001

**Fig. 5:**
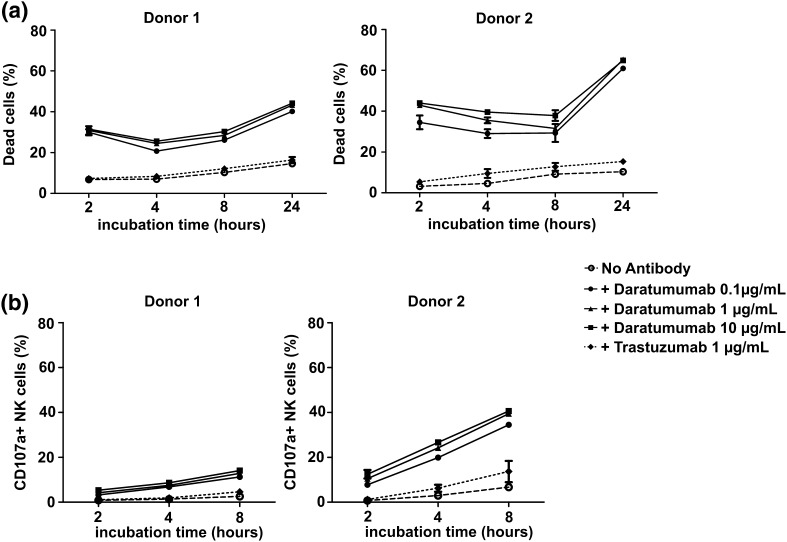
The kinetics of NK cell death and activation in the absence or presence of daratumumab. IL-2 activated NK cells were incubated with or without 0.1, 1, 10 µg/mL daratumumab or 1 µg/mL trastuzumab for 2, 4, 8, or 24 h in 21% O_2_. **a** The percentage of dead NK cells was calculated by the percentage of NK cells positive for Live/Dead^®^ Marker. **b** NK cell degranulation was determined as the percentage of CD107a+ NK cells. Shown are dots representing the mean of replicate cultures with standard deviation

NK cells also express low levels of CD38 which potentially binds daratumumab and therefore could be a target of daratumumab-mediated killing. Since reducing the number of effector cells could be detrimental, we also evaluated whether there was an increase in the number of dead NK cells after addition of daratumumab. In the absence of daratumumab and tumor cells, the average percentage of dead NK cells after 4 h of culture was 21.49% compared with 36.66% in the presence of daratumumab (Fig. 4a). This phenomenon was also observed in the presence of TMEF. An increase in the percentage of dead NK cells in conditions with daratumumab was also observed after 4 h co-culture with the different tumor cell as well as in the presence of TMEF lines (*p* < 0.0001, Fig. 4b). An increased NK cell death by daratumumab was observed after only 2 h and cell death further increased to 60% after 24 h (Fig. 5a). Induction of NK cell death was not observed with trastuzumab which binds Her2/neu that is not present on NK cells. Furthermore, the effect of daratumumab was comparable for conditions with 0.1, 1 and 10 μg/mL of antibody. We also observed a higher percentage of CD107a positive NK cells in all conditions with daratumumab. This demonstrates that NK cells increasingly degranulate upon the addition of daratumumab, suggesting that they get activated by- and mediate ADCC against-other NK cells with daratumumab bound to their surface (Fig. 5b).

### Selection of KIR-ligand mismatched donors can help to potentiate NK cell anti-MM reactivity in the TME

**Fig. 6:**
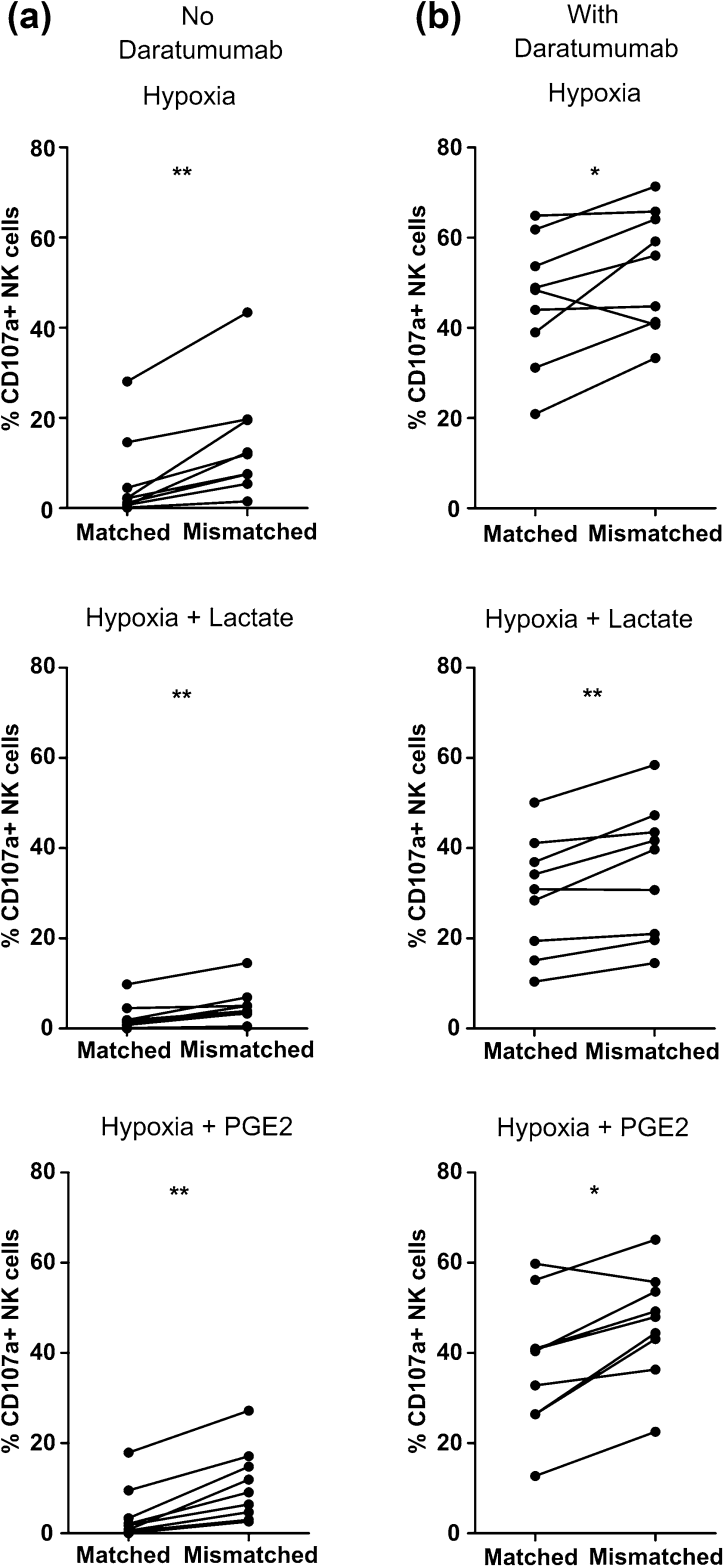
Comparison of degranulation of KIR-ligand mismatched NK cells and KIR-ligand matched NK cells in response to MM cells with or without daratumumab. Following an overnight incubation under hypoxia (0.6% O_2_), UM9 and RPMI8226/s cells were incubated **a** without or **b** with daratumumab for 30 min while IL-2 activated NK cells were incubated for 1 h with PGE2 or lactate. The percentage of degranulating KIR-ligand matched or KIR-ligand mismatched NK cells was determined as % CD107a+ cells. Dots represent means of replicate cultures of independent donors. *n* = 5 independent experiments

After showing that triggering ADCC is a potent way to enhance NK cell anti-MM reactivity in the presence of TMEF, we questioned whether the selection of KIR-ligand mismatched donors could help to further augment the response. We performed a CD107a assay and determined the degranulation of NKG2A negative NK cells (KIR-ligand matched vs mismatched subsets) against CD38-high UM9 or RPMI8226/s cells under different TMEFs and in the absence or presence of daratumumab. The addition of daratumumab to the culture enhanced NK cell degranulation of both KIR-ligand matched and mismatched NK cell subsets upon co-culture with UM9 or RPMI8226/s as compared to conditions without daratumumab (Fig. 6 and supplementary Fig. 5). For KIR-ligand matched NK cells, the average increase by daratumumab was from 6.03 to 45.87% under hypoxia (*p* = 0.0039), 2.34–29.62% under hypoxia and lactate (*p* = 0.0039), and 4.05–37.38% under hypoxia and PGE2 (*p* = 0.0039). For KIR-ligand mismatched NK cells, the average increase was from 14.32 to 52.96% under hypoxia (*p* = 0.0039), 4.76–35.17% under hypoxia and lactate (*p* = 0.0039), and 10.76–46.44% under hypoxia and PGE2 (*p* = 0.0039). We did not observe a difference in the percentage of NK cells degranulating spontaneously or in response to HLA class I negative K562 cells for subsets single positive for KIR2DL1, KIR2DL2/3 or KIR3DL1 (supplementary Fig. 6 and supplementary Fig. 7).

In the absence of daratumumab, the average percentage of degranulating NK cells was higher for the KIR-ligand mismatched subset as compared to the matched subset for all donors tested in response to UM9 or RPMI8226/s. This was observed under hypoxia (*p* = 0.0039); hypoxia and lactate (*p* = 0.0091); and hypoxia and PGE2 (*p* = 0.0091) (Fig. 6a). In the presence of daratumumab, there was little difference between degranulation of the KIR-ligand matched and mismatched subsets (Fig. 6b). This suggests that lowering the activation threshold by KIR-ligand mismatching would be most effective under conditions where the NK cell receives limited activating signals. Furthermore, for both the KIR-ligand mismatched as well as the matched subset, the percentage of degranulating NK cells in response to UM9 or RPMI8226/s with daratumumab was not significantly different between the TME conditions.

## Discussion

In this study, we set out to explore whether the combination of alloreactive NK cells and clinical antibodies targeting tumor-specific/-associated antigens could help to overcome the detrimental effects of the immunosuppressive TME. First, we demonstrated that the NK cell antitumor response can be potentiated by clinical-grade antibodies and that this was effective even under conditions reflective of an NK cell suppressive TME. Additionally, we provide data demonstrating that selection of KIR-ligand mismatched NK cell donors could help to amplify the NK cell response further.

In our previous study, we showed that hypoxia alone could inhibit NK cell anti-MM activity and we demonstrated that IL-2 activation of NK cells could overcome this issue [4]. In this study, we aimed to investigate the influence of a more severe NK-suppressive TME. We observed the inhibitory effect of lactate on NK cell killing already at a low (5 mM) concentration (supplementary Fig. 1) which was in line with earlier studies [7]. PGE2 has been shown to negatively impact the NK cell antitumor response [6]. In our hands, NK cell inhibition by PGE2 was less pronounced than with lactate and seemed to be more cell line dependent. In a mouse myeloma model, oxygen levels of less than 10 mmHg (1.3%) have been shown [24]. In patients with MM, the accumulation of hypoxia-inducible factor-1α (HIF-1α) in bone marrow (BM) biopsies suggested the presence of a hypoxic region in the BM [25, 26] and in other tumors, hypoxia frequently coincides with elevated lactate levels. Exact lactate and PGE2 levels in MM BM should, however, be determined to confirm the relevance of these factors for MM. Another important point to consider in more detail in the future is tumor heterogeneity. Although we already performed our assays using multiple cell lines, it is necessary to perform follow-up studies with more heterogeneous primary myeloma cells. In vivo, the tumor and the TME will be more complex and potentially more immunosuppressive which necessitates additional clinical studies in human patients. Nevertheless, our study already illustrated that IL-2 activation alone was not enough to potentiate NK cells which clearly emphasized the need for further activation of NK cells to overcome the inhibitory effect of multiple TMEFs.

In this study, we demonstrated that daratumumab enhanced NK cell-mediated killing of cancer cells under hypoxia, hypoxia/lactate, and hypoxia/PGE2. In line with a previous study [27], we showed that the effect of daratumumab was specific for MM lines expressing relatively high levels of CD38 (i.e., UM9 and RPMI8226/s). We did not observe ADCC in response to CD38 low or negative cells, suggesting that the level of antigen expression on target cells could affect the outcome. Our observation that CD38 expression by MM cells was not reduced by TMEFs, and that binding of daratumumab was not affected by any of the TME conditions, was critical since CD38 expression was required for daratumumab to be effective. Previously, we and others showed that hypoxia alone could slightly reduce CD16 expression [4, 5]. Here, we did not observe a decrease in ADCC capacity under hypoxia alone (Fig. 3), so apparently, this lower CD16 expression did not influence the capacity of NK cells to mediate ADCC. In line with our TME data, daratumumab has previously been shown to potentiate PBMC mediated anti-MM reactivity in the presence of immunosuppressive BM stromal cells and in a mouse model of MM [28]. Together with our current data, this emphasizes the potency of an approach using an ADCC triggering antibody to potentiate NK cells in the TME.

The current availability of a large number of clinically available antibodies which bind to tumor-specific/associated antigens (e.g., cetuximab, rituximab, trastuzumab) on a variety of tumors provides the great opportunity to combine NK cell and antibody therapies. A major challenge, however, is to find antibodies that exclusively bind to tumor cells. Antibodies, such as daratumumab, also interact with antigens expressed on healthy cells, and could give rise to off-target cytotoxicity. We indeed observed a high number of dead or degranulating NK cells in conditions where daratumumab but no tumor target cells were present. Supporting this, in a previous study in MM patients, administration of daratumumab has been shown to decrease the number of peripheral blood NK cells [29, 30]. These clinical data and our current data could be explained by the fact that NK cells express CD38 [31]. Binding of daratumumab to CD38+ NK cells could trigger ADCC of NK cells against NK cells with bound daratumumab, a phenomenon called fratricide which would be in line with our degranulation data. Elotuzumab, an antibody against CS1 expressed on NK cells and MM cells, has been shown to activate NK cells directly [29]. Presumably, this is not the mechanism for daratumumab as a CD38-F(ab’)_2_ fragment did not trigger direct NK cell activation [32]. A reduction in NK cell numbers, upon administration to patients, could be detrimental on the long term and the treatment can possibly be further optimized by incubating NK cells, before infusion, with a CD38-F(ab’)_2_ fragment to reduce binding of the ADCC mediating antibody.

KIR-ligand mismatched NK cells were more effective against MM target cells than matched cells under all TME conditions tested in this study. Degranulation of both subsets was enhanced by daratumumab, and although the difference between matched and mismatched NK cells was less distinct upon the addition of daratumumab, mismatched NK cells seemed to be slightly better effector cells. The difference between matched and mismatched cells was not caused by intrinsic differences between the subsets as, in response to HLA class I negative K562 cells, all subsets degranulated to the same extent (supplementary Fig. 7). KIR/HLA interactions have been shown to reduce ADCC mediated by rituximab [16, 19, 20, 33]. One of these studies showed that inhibitory KIR/HLA interactions could be compensated for by modifying the Fc part of the rituximab antibody by means of glycoengineering resulting in an antibody called GA101 (obinutuzumab) with an enhanced potential to trigger ADCC [33]. An alternative approach that has been proposed is to use the KIR blocking antibodies that are currently available in the clinical-grade format [20]. Unfortunately, a recent study showed that the administration of anti-KIR antibody in patients with smoldering MM had to be terminated as it resulted in NK cell anergy caused by the removal of KIR receptor from NK cell surface by trogocytosis [34]. In the current study, we demonstrate that selection of a donor based on HLA genotype and KIR expression could be a good way to achieve a KIR-ligand mismatched status to minimize the detrimental effects of KIR/HLA. Selection of KIR-ligand mismatched donors would be feasible for allogeneic NK cell treatments where the patient lacks at least one of the HLA epitopes binding to inhibitory KIRs, as is the case in approximately 70% of the individuals (Mahaweni unpublished data and [35]). Also in the situation where a glycoengineered antibody is used, selection of a KIR-ligand mismatched donor could be beneficial since tumor cells could downregulate the expression of the antigen targeted by the antibody. In that case, the KIR-ligand mismatch will still facilitate the response against antigen negative cells.

In summary, we showed in this study that the combination of an ADCC triggering antibody and selection of KIR-ligand mismatched donors is a potent and realistic platform to potentiate the NK cell antitumor response in the TME. The difference between allogeneic and non-allogeneic NK cells has to be explored in vivo. The antitumor potential of NK cells can be applied by donor NK-cell infusion as well as by haploidentical stem-cell transplantation (HaploSCT). In HaploSCT, donor-derived NK cells have been identified as the main mediators of antitumor reactivity, and due to the improved post-transplant treatment regimen, HaploSCT is now a realistic and feasible treatment option. Therefore, we envision that the combination of ADCC triggering antibodies and KIR-ligand mismatching is a favorable combination to be tested in future clinical studies, both in the context of HaploSCT, potentially in combination with NK cell infusions, as well as single donor NK-cell infusion.

## Electronic supplementary material

Below is the link to the electronic supplementary material.

Supplementary material 1 (PDF 851 KB)

## Abbreviations

ADCC: Antibody-dependent cell-mediated cytotoxicity
BM: Bone marrow
CS-BLI: Compartment specific-bioluminescence imaging
FCS: Fetal calf serum
HaploSCT: Haploidentical stem cell transplantation
HLA: Human leukocyte antigen
IL-2: Interleukin 2
KIR: Killer immunoglobulin-like receptor
mAbs: Monoclonal antibodies
MM: Multiple myeloma
NK cell: Natural killer cell
PBMC: Peripheral blood mononuclear cell
PGE2: Prostaglandin E2
TME: Tumor microenvironment
TMEFs: Tumor microenvironmental factors

## References

[1] Vivier E, Ugolini S, Blaise D (2012). Targeting natural killer cells and natural killer T cells in cancer. Nat Rev Immunol.

[2] Sun Y (2016). Tumor microenvironment and cancer therapy resistance. Cancer Lett.

[3] Junttila MR, de Sauvage FJ (2013). Influence of tumour micro-environment heterogeneity on therapeutic response. Nature.

[4] Sarkar S, Germeraad WTV, Rouschop KMA (2013). Hypoxia induced impairment of NK cell cytotoxicity against multiple myeloma can be overcome by IL-2 activation of the NK cells. PLoS One.

[5] Balsamo M, Manzini C, Pietra G (2013). Hypoxia downregulates the expression of activating receptors involved in NK-cell-mediated target cell killing without affecting ADCC. Eur J Immunol.

[6] Pietra G, Manzini C, Rivara S (2012). Melanoma cells inhibit natural killer cell function by modulating the expression of activating receptors and cytolytic activity. Cancer Res.

[7] Husain Z, Huang Y, Seth P, Sukhatme VP (2013). Tumor-derived lactate modifies antitumor immune response: effect on myeloid-derived suppressor cells and NK cells. J Immunol.

[8] Brand A, Singer K, Koehl GE (2016). LDHA-associated lactic acid production blunts tumor immunosurveillance by T and NK cells. Cell Metab.

[9] Wang W, Guo H, Geng J (2014). Tumor-released galectin-3, a soluble inhibitory ligand of human NKp30, plays an important role in tumor escape from NK cell attack. J Biol Chem.

[10] Kopp H-G, Placke T, Salih HR (2009). Platelet-derived transforming growth factor-down-regulates NKG2D thereby inhibiting natural killer cell antitumor reactivity. Cancer Res.

[11] Viel S, Marcais A, Guimaraes FS-F (2016). TGF- inhibits the activation and functions of NK cells by repressing the mTOR pathway. Sci Signal.

[12] Hoechst B, Voigtlaender T, Ormandy L (2009). Myeloid derived suppressor cells inhibit natural killer cells in patients with hepatocellular carcinoma via the NKp30 receptor. Hepatology.

[13] Mao Y, Sarhan D, Steven A (2014). Inhibition of tumor-derived prostaglandin-E2 blocks the induction of myeloid-derived suppressor cells and recovers natural killer cell activity. Clin Cancer Res.

[14] Bryceson YT, March ME, Ljunggren H-G, Long EO (2006). Synergy among receptors on resting NK cells for the activation of natural cytotoxicity and cytokine secretion. Blood.

[15] Seidel UJE, Schlegel P, Lang P (2013). Natural killer cell mediated antibody-dependent cellular cytotoxicity in tumor immunotherapy with therapeutic antibodies. Front Immunol.

[16] Wang W, Erbe AK, Hank JA (2015). NK cell-mediated antibody-dependent cellular cytotoxicity in cancer immunotherapy. Front Immunol.

[17] Nijhof IS, Groen RWJ, Noort WA (2015). Preclinical evidence for the therapeutic potential of CD38-targeted immuno-chemotherapy in multiple Myeloma patients refractory to Lenalidomide and Bortezomib. Clin Cancer Res.

[18] Sarkar S, van Gelder M, Noort W (2015). Optimal selection of natural killer cells to kill myeloma: the role of HLA-E and NKG2A. Cancer Immunol Immunother.

[19] Kohrt HE, Thielens A, Marabelle A (2014). Anti-KIR antibody enhancement of anti-lymphoma activity of natural killer cells as monotherapy and in combination with anti-CD20 antibodies. Blood.

[20] Binyamin L, Alpaugh RK, Hughes TL (2008). Blocking NK cell inhibitory self-recognition promotes antibody-dependent cellular cytotoxicity in a model of anti-lymphoma therapy. J Immunol.

[21] McMillin DW, Delmore J, Weisberg E (2010). Tumor cell-specific bioluminescence platform to identify stroma-induced changes to anticancer drug activity. Nat Med.

[22] Walenta S, Wetterling M, Lehrke M (2000). High lactate levels predict likelihood of metastases, tumor recurrence, and restricted patient survival in human cervical cancers. Cancer Res.

[23] Hidalgo GE, Zhong L, Doherty DE, Hirschowitz E (2002). Plasma PGE-2 levels and altered cytokine profiles in adherent peripheral blood mononuclear cells in non-small cell lung cancer (NSCLC). Mol Cancer.

[24] Hu J, Van Valckenborgh E, Menu E (2012). Understanding the hypoxic niche of multiple myeloma: therapeutic implications and contributions of mouse models. Dis Model Mech.

[25] Martin SK, Diamond P, Williams SA (2010). Hypoxia-inducible factor-2 is a novel regulator of aberrant CXCL12 expression in multiple myeloma plasma cells. Haematologica.

[26] Giatromanolaki A, Bai M, Margaritis D (2010). Hypoxia and activated VEGF/receptor pathway in multiple myeloma. Anticancer Res.

[27] Sanchez L, Wang Y, Siegel DS, Wang ML (2016). Daratumumab: a first-in-class CD38 monoclonal antibody for the treatment of multiple myeloma. J Hematol Oncol.

[28] de Weers M, Tai Y-T, van der Veer MS (2011). Daratumumab, a novel therapeutic human CD38 monoclonal antibody, induces killing of multiple myeloma and other hematological tumors. J Immunol.

[29] Phipps C, Chen Y, Gopalakrishnan S, Tan D (2015). Daratumumab and its potential in the treatment of multiple myeloma: overview of the preclinical and clinical development. Ther Adv Hematol.

[30] McEllistrim C, Krawczyk J, O’Dwyer ME (2017). New developments in the treatment of multiple myeloma—clinical utility of daratumumab. Biologics.

[31] Krejcik J, Casneuf T, Nijhof IS (2016). Daratumumab depletes CD38+ immune regulatory cells, promotes T-cell expansion, and skews T-cell repertoire in multiple myeloma. Blood.

[32] Cherkasova E, Espinoza L, Kotecha R, Reger RN, Berg M, Aue G, Attar RM, Sasser AK, Carlsten M, Childs RW (2015). Treatment of ex vivo expanded NK cells with daratumumab F(ab’)2 fragments protects adoptively transferred NK cells from daratumumab-mediated killing and augments daratumumab-induced antibody dependent cellular toxicity (ADCC) of myeloma. Blood.

[33] Terszowski G, Klein C, Stern M (2014). KIR/HLA Interactions negatively affect rituximab—but not GA101 (Obinutuzumab)-induced antibody-dependent cellular cytotoxicity. J Immunol.

[34] Carlsten M, Korde N, Kotecha R (2016). Checkpoint inhibition of KIR2D with the monoclonal antibody IPH2101 induces contraction and hyporesponsiveness of NK cells in patients with myeloma. Clin Cancer Res.

[35] Omar SYA, Alkuriji A, Alwase S (2016). Genotypic diversity of the killer cell immunoglobulin-like receptors (KIR) and their HLA class I ligands in a saudi population. Genet Mol Biol.

