# Supplementary material for: Daratumumab augments alloreactive natural killer cell cytotoxicity towards CD38+ multiple myeloma cell lines in a biochemical context mimicking tumour microenvironment conditions
Source: Cancer Immunol Immunother. 2018 Mar 2;67(6):861–72. doi: 10.1007/s00262-018-2140-1 (PMC5951903; doi:10.1007/s00262-018-2140-1)
Supplement: Supplementary file 1 — Supplementary material 1 (PDF 851 KB) [file 262_2018_2140_MOESM1_ESM.pdf]

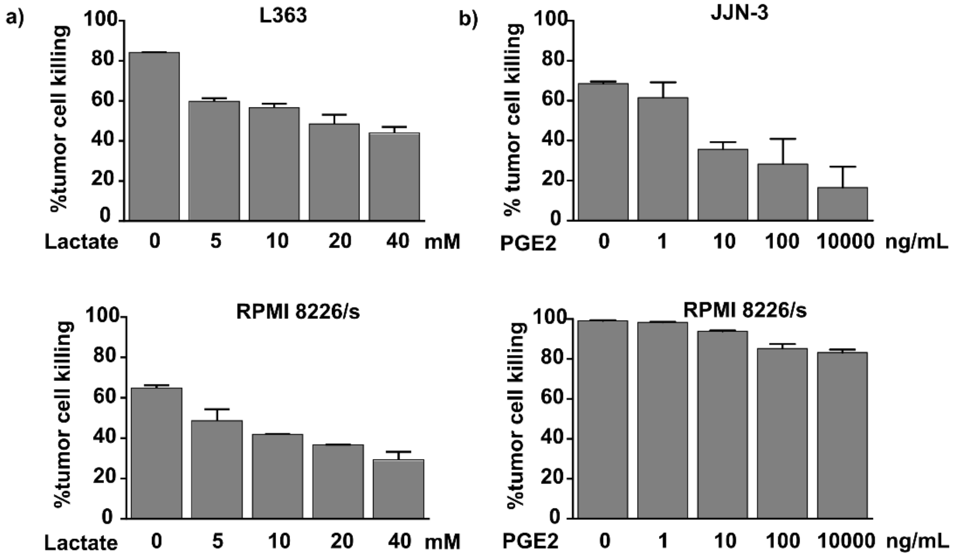

Supplementary figure 1. Effect of lactate and PGE2 on the killing capacity of NK cells.
In a compartment specific bioluminescence imaging (CS-BLI) based assay, luciferase-expressing L363, JJN-3 or RPMI8226/s cells were incubated overnight with KHYG-1 NK cell line, in the absence or presence of indicated concentrations of **a** lactate or **b** PGE2. Shown are the mean and standard deviation (SD) of n =4 technical replicates from one experiment.

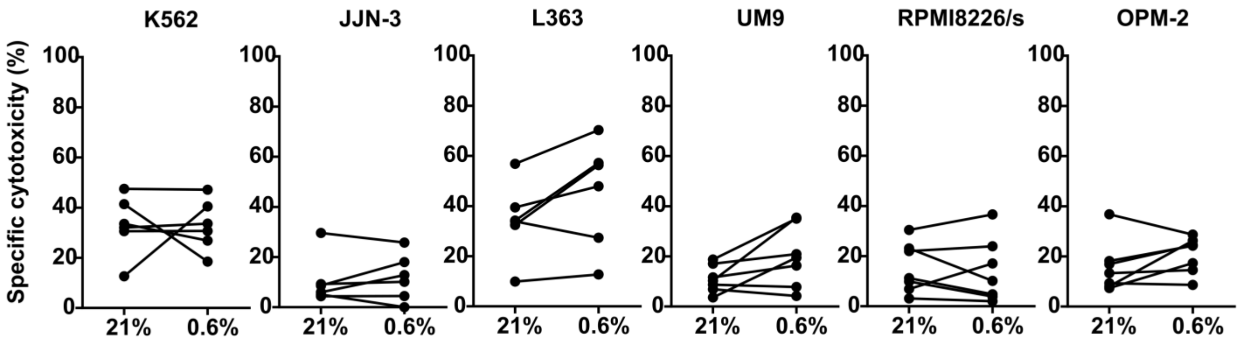

Supplementary figure 2. Killing of tumor cells by IL-2-activated NK cells under normoxia and hypoxia.

IL-2 activated NK cells were co-cultured with DiI-labeled target cells in 1:1 E:T ratio in a 4 hour flow cytometry-based cytotoxicity assay, under 21% O_2 (ambient air) or 0.6% O_2 (hypoxia). Each dot represents the mean of replicate culture of one donor (n = 5 donors in 5 independent experiments).

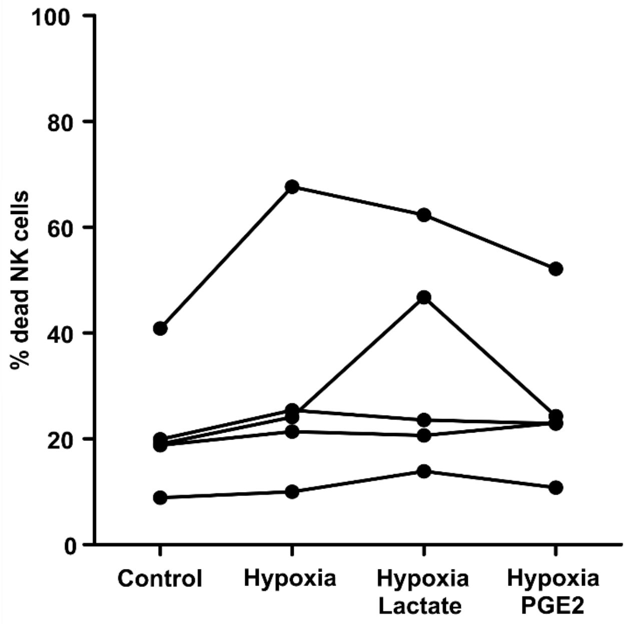

Supplementary figure 3. Percentage of dead NK cells exposed to TME factors.

NK cells were cultured under 21% O_2 only (control), or under hypoxia (0.6% O_2) or in combination of hypoxia with lactate or PGE2 for 5 hours. Dead cells were evaluated by calculating the percentage of NK cells positive for Live/Dead Marker. Per condition, each dot represents the mean of replicate culture of one donor (n = 5 donors in 5 independent experiments).

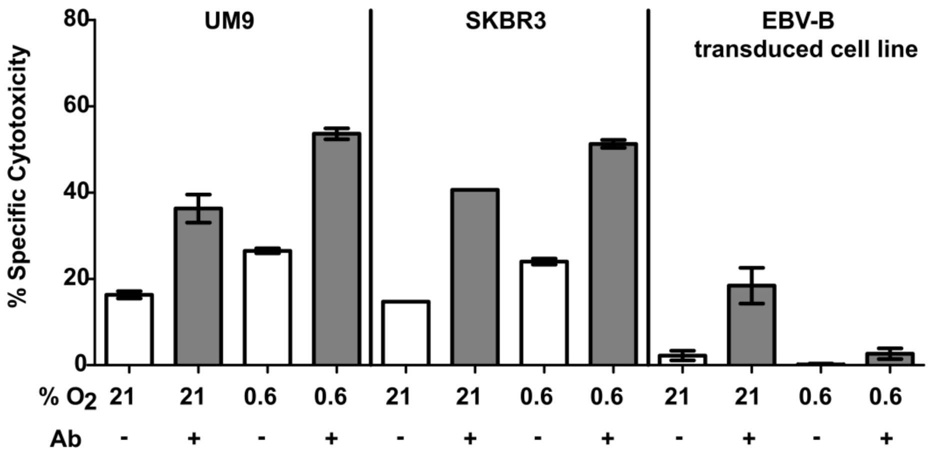

Supplementary figure 4. Effect of clinical-grade antibodies on NK cell mediated ADCC under hypoxia.

DiI-labeled UM9 (CD38+), SKBR3 (HER2+), or EBV-B transduced cell line (CD20+) were incubated overnight at 0.6% or 21% O_2. The next day, 30 minutes prior to 4 hours flow cytometry-based cytotoxicity assay, tumor cells were incubated with either 1 μg/mL daratumumab (UM9), 1 μg/mL trastuzumab (SKBR3), or 10 μg/mL rituximab (EBV-B transduced cell line) while IL-2 activated NK cells were incubated for 1 hour with 100 ng/mL PGE2 or 50 mM lactate. Shown are the mean of replicate culture with SD. n = 2 experiments for UM9 and SKBR3, 1 experiment for EBV-B

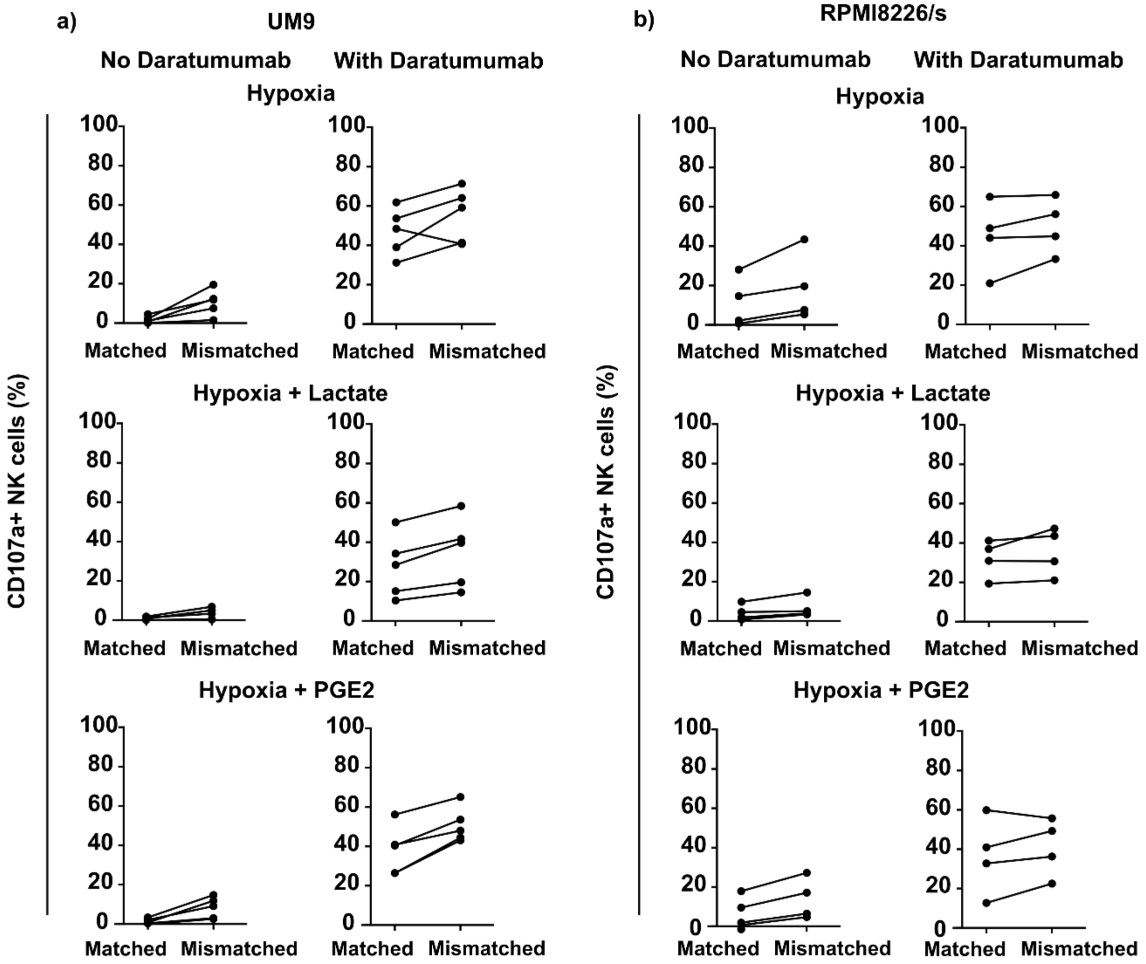

Supplementary figure 5. Comparison of degranulation of KIR-ligand mismatched NK cells and KIR-ligand matched NK cells in response to MM cells with or without daratumumab.

Following an overnight incubation in the presence of hypoxia (0.6% O_2), **a** UM9 and **b** RPMI8226/s cells were incubated with daratumumab for 30 minutes while IL-2 activated NK cells were incubated for 1 hour with 100 ng/mL PGE2 or 50 mM lactate.

Dots represent means of replicate cultures. n = 5 independent experiments

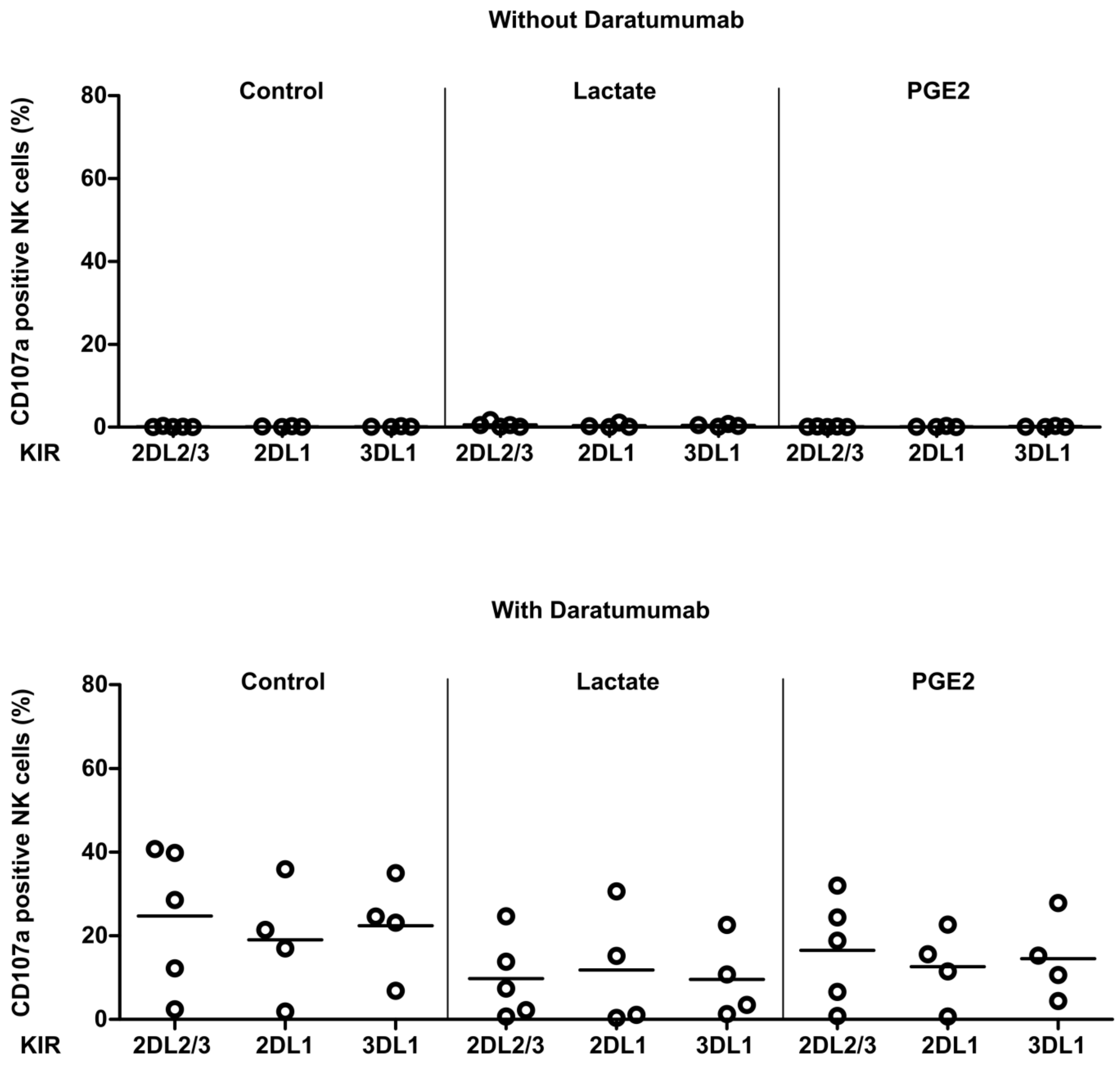

Supplementary figure 6. Spontaneous degranulation of NK cell subsets

NK cell spontaneous degranulation was measured in a flow cytometry-based degranulation (CD107a) assay after 5 hours incubation of NK cells under hypoxia (0.6% O_2) alone (control) or the combination of hypoxia and lactate or hypoxia and PGE2 in the presence or absence of daratumumab. Each dot represents the mean of replicate culture of one donor (n = 5 donors in 5 independent experiments).

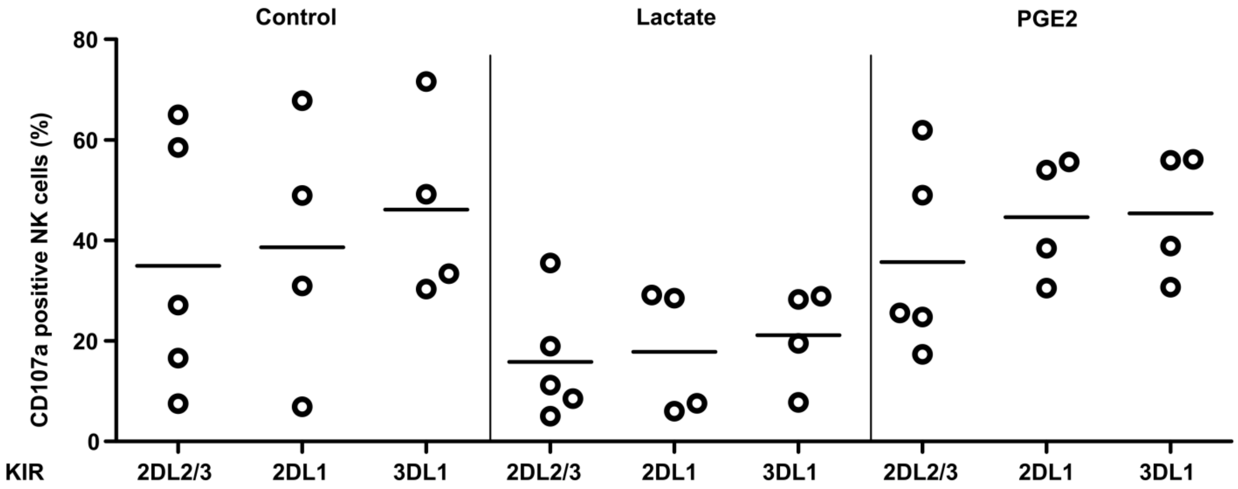

Supplementary figure 7. Degranulating capacity of KIR2DL2/3, KIR2DL1, and KIR3DL1 subsets in the absence of inhibitory signals from HLA class I.

NK cells were co-cultured with K562 cells, in a flow cytometry-based degranulation (CD107a) assay in the presence of hypoxia (0.6% O_2) alone (control) or the combination of hypoxia and lactate or hypoxia and PGE2. Each dot represents the mean of replicate culture of one donor (n = 5 donors in 5 independent experiments).
